# HER2-targeted therapy in colorectal cancer: a comprehensive review

**DOI:** 10.1007/s12094-025-03887-0

**Published:** 2025-03-14

**Authors:** Yeliz Benli, Helin Arıkan, Özge Akbulut-Çalışkan

**Affiliations:** Department of Molecular Biology and Genetics, Faculty of Science and Letters, Başkent University, 06790 Ankara, Turkey

**Keywords:** HER2, Colorectal cancer, Cancer therapeutics, Targeted therapy, Metastasis

## Abstract

Colorectal cancer (CRC) is the third most common cancer and the second leading cause of cancer-related deaths worldwide. Despite treatment advancements in the last decades, CRC remains heterogeneous with significant clinical and genetic diversity. Human epidermal growth factor receptor 2 (*HER2*) proto-oncogene plays a critical role, as its amplification or overexpression leading to abnormal cell proliferation and tumorigenesis. *HER2* overexpression or amplification is identified in 2–4% of metastatic CRCs (mCRC) patients, representing a potential therapeutic target. It is also associated with resistance against epidermal growth factor receptor (EGFR)-targeted therapies like cetuximab and panitumumab, for treatment of *RAS* wild-type mCRC. Although HER2-positive mCRC is rare, assessing HER2 levels is important. Furthermore, anti-HER2 therapies exhibited non-toxic profile and high efficacy in chemorefractory cases. This review delves into modern management of anti-HER2 therapies in CRC with a particular focus on recent advances and current knowledge about the prognostic and predictive value of HER2.

## Introduction

Colorectal cancer (CRC) ranks as the third most commonly diagnosed cancer and the second most common cause of cancer malignancy-related mortality worldwide [1]. CRC stands as a global health problem with 1.9 million cases per year. In the USA, an estimated 152,810 people will be diagnosed with CRC in 2024, and 53,010 will die from it [1]. It is a heterogeneous disease, exhibiting clinical and genetic diversity, and is influenced by multiple signaling pathways. It typically originates from several gene related events including epigenetic alterations or mutations which may transform a normal glandular epithelium of colon or rectum into a benign neoplasm [2]. Especially, a family history of CRC may account for up to 25% of cases, indicating a potential genetic component as first-degree relatives (FDRs) with a family history of CRC have a 2- to fourfold increased risk of getting the disease [3].

Despite significant advances on efficacious therapeutics and useful diagnostic approaches for early detection of CRC, approximately 33% of colorectal malignancies develop metastases at peritoneum or more distant organs such as lung, bone, brain, and liver [4–6]. Unfortunately, the 5-year overall survival (OS) rate for patients with distant metastatic CRC (mCRC) is approximately 15% (SEER 22, NCI). Still, it remains a highly lethal disease with approximately 30% of patients experiencing a recurrence or metastases even after undergoing initial curative surgical treatment or receiving adjuvant chemotherapy [7].

Due to heterogeneity of the patient population and different molecular subtypes, assessing a suitable treatment option is difficult. Surgery followed by radiotherapy, radiofrequency ablation, cryosurgery, chemotherapy, and targeted therapy are the therapeutic options for the treatment of CRC. Among these, chemotherapy is considered a crucial therapeutic approach, especially for patients in advanced stages who are unable to undergo surgery, which may be followed by a specific targeted therapy [8]. For example, chemotherapy and anti–epidermal growth factor receptor (EGFR) (i.e., panitumumab and cetuximab) therapy is recommended for *RAS* wild-type mCRC patients with microsatellite stable (MSS) or proficient mismatch repair mechanism. Moreover, pembrolizumab, an immune checkpoint inhibitor as targeted therapy, is recommended for mCRC patients with microsatellite instability-high (MSI-H) or deficient mismatch repair tumors [9, 10].

Additionally, amplification or overexpression of the human epidermal growth factor receptor 2 (*HER2*) gene has garnered significant attention in the field of cancer research as a novel oncotarget in mCRC [9, 10]. In this review, we point out the modern management of anti-HER2 therapies in CRC with a particular focus on recent advances and current knowledge about the prognostic and predictive value of HER2.

## *HER2* as an oncogenic driver in colorectal cancer

**Fig. 1:**
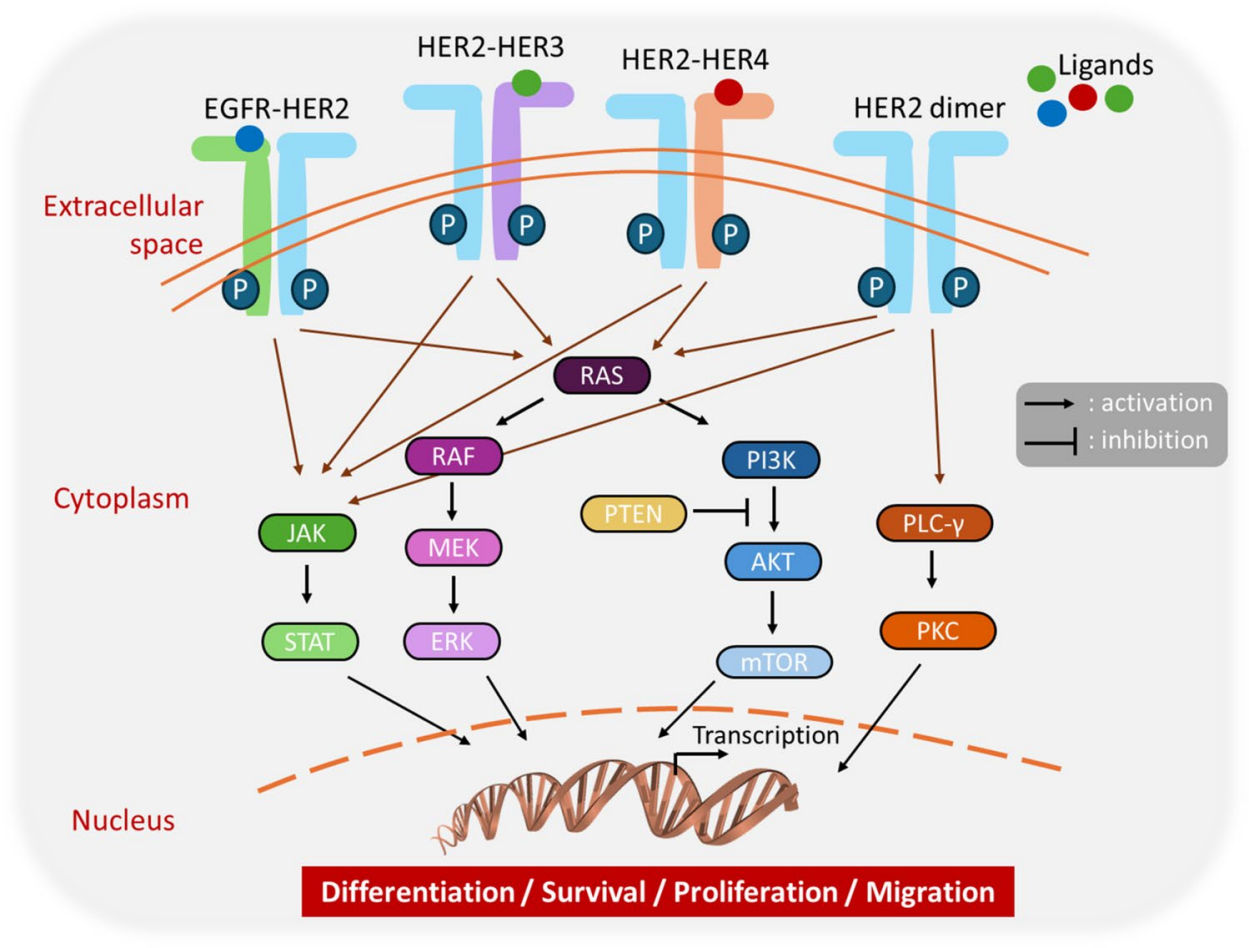
The schematic representation of physiological roles and mechanism of action of HER2 in CRC through several signaling pathways. Upon HER2 dimerization with other family members or itself leads to activation of downstream signaling events including pathways like MAPK, PI3K/AKT, JAK/STAT, and PLC/PKC. The signaling culminates in transcriptional changes affecting cellular differentiation, survival, proliferation, and migration

*HER2* is a proto-oncogene located on chromosome 17q21 and is a member of ERBB family of receptor tyrosine kinases [11]. HER2 does not bind any ligands but can form homo- or hetero-dimers with other EGFR family members (HER1/EGFR, HER3, HER4). This triggers transphosphorylation of tyrosine kinase domain, activating key signaling pathways such as phosphoinositide 3 kinase (PI3K)/Akt/mammalian target of rapamycin (mTOR), transducer and activator of transcription (JAK/STAT) pathway, RAS-RAF-mitogen-activated protein kinase/extracellular signal-regulated kinase (MAPK/ERK) pathway, and phospholipase C (PLC)/protein kinase C (PKC) pathway. These pathways regulate cell survival, proliferation, and migration [12–14]. *HER2* oncogene amplification or overexpression of its protein causes self-dimerization and hyperactivation of mitotic signals. This initiates a series of parallel signaling cascades that ultimately cause phenotypic changes in cell behavior and tumorigenesis [15]. Figure 1 summarizes the receptor-ligand binding (except for HER2 receptor) and the signaling pathways enrolled in CRC cells.

*HER2* gene overexpression or amplification identifies a specific subset of colorectal malignancies, which is observed in approximately 2–4% of patients with mCRC, and represents a rapidly emerging therapeutic target in these patients [12]. According to a meta-analysis of clinical outcomes, patients diagnosed with HER2-positive mCRC exhibited a substantially elevated risk of mortality or disease advancement, approximately 2.84 times greater than that observed in patients with HER2-negative mCRC [16]. Even, advanced-stage cancers with a high burden of genetic mutations are more likely to demonstrate overexpression of HER2 [17].

Previous research demonstrated a high occurrence of *HER2* amplification in left-sided CRC patients, and that HER2-positive CRCs are mutually exclusive with mutations in *KRAS*, *NRAS*, and *BRAF* oncogenic drivers [18, 19]. For instance, KRAS alterations (27.3%) were found to be significantly less common in *HER2*-amplified CRC samples compared to wild-type versions (51.8%) [20]. Moreover, Raghav et al., showed that 37 of 99 mCRC patients carrying *BRAF* and *KRAS* wild-type genes had *HER2* amplification [21]. However, it should be noted that a small subset of tumors may harbor co-occurring mutations on these genes [20]. This is also correlated with a higher frequency of metastases to distant organs such as lung, peritoneal, and brain [19, 22, 23].

In addition to being amplified, *HER2* somatic mutations can also be detected in CRC at a frequency of 4%. These mutations may sometimes occur together with *HER2* amplification or alterations in other oncogenes such *RAS*, *BRAF,* and *EGFR* [18]. Similar to *HER2* gene amplification, *HER2* activating mutations (more frequently, V841I, S310F, L755S, V777L, and I655V) affect the extracellular, transmembrane, or cytoplasmic domain which in turn cause hyperactivation of proliferative signals [24, 25]. Moreover, in a recent study, another *HER2* mutation (G776S) functions as an oncogenic driver and triggers *HER2* downstream signaling when accompanied by a tumor suppressor gene, *APC*, loss-of-function mutations [26]. CRC cell xenografts with the *HER2* G776S mutation were treated with a pan-HER tyrosine kinase inhibitor, afatinib, leading to significantly reduced tumor growth compared to control-treated mice [26]. Remarkably, a 78-year-old woman with mCRC with a *HER2* L726L mutation, who does not have HER2 amplification or overexpression, showed an excellent clinical response to fam-trastuzumab deruxtecan (T-DXd) [27]. This notable case also highlights the significance of comprehensive genomic testing in the context of metastatic cancers. These preclinical and clinical findings support the need for conducting more clinical trials targeting *HER2* activating mutations in mCRC patients, even without HER2 amplification.

## HER2 as a predictive biomarker for resistance to anti-EGFR therapies in mCRC

Considering HER2 heterogeneity as a prognostic marker, HER2 amplification has also been identified as a key mechanism driving resistance to anti-EGFR therapies in CRC by sustaining downstream signaling [28]. EGFR-targeted therapies like cetuximab and panitumumab are being used for the treatment of mCRC patients who are wild-type for *RAS* gene [29]. Unfortunately, the effectiveness and clinical practicality of targeted therapy are hindered by the development of resistance. Especially, in *RAS/RAF* wild-type mCRC patients, HER2 amplification or overexpression can cause resistance to these anti-EGFR therapies, making it a predictive of treatment failure [16, 30]. Therefore, conducting an HER2 amplification screen ahead of anti-EGFR therapies can guide therapy decisions to benefit most. Bertotti et al., conducted a multi-arm study in *HER2*-amplified patient-derived xenografts (PDXs) derived from mCRC patient samples and showed that simultaneous inhibition of HER2 and EGFR resulted in significant and sustained tumor regression [31]. Particularly, a *HER2*-amplified cetuximab-resistant CRC xenograft model was tested against the dual EGFR/HER2 tyrosine kinase inhibitor, lapatinib, and anti-HER2 medication pertuzumab, with or without cetuximab. Lapatinib and cetuximab combinations resulted in a significant and long-lasting tumor shrinkage, while pertuzumab alone or in combination with cetuximab only caused a negligible delay in tumor growth [31]. Subsequently, a small-sample clinical study confirmed the presence of the similar response among mCRC patients [32].

As a significant molecular marker in cancer, *BRAF* mutations can activate the RAF/MAPK pathway without the need of EGFR activation, causing a limited response to cetuximab [33]. Resistance to BRAF inhibitors can arise due to the compensatory activation of the MAPK pathway, occurring as a result of the acquisition or preexistence HER2 receptor tyrosine kinase amplification [34, 35]. This molecular event undermines the efficacy of BRAF inhibitors in the treatment of CRC.

Not only HER2 amplification but also *HER2* activating mutations (S310F, L755S, V777L, V842I, and L866M) cause oncogenic transformations of colon epithelial cells and induce drug resistance to anti-EGFR therapies, cetuximab and panitumumab in CRC cell lines [25]. Likewise, PDXs with *HER2* mutations retained their tumor regression after dual treatment of anti-HER2 therapies (trastuzumab plus neratinib or trastuzumab plus lapatinib) compared to single agents [25, 36].

## Emerging anti-HER2 therapies for HER2-positive CRC

**Fig. 2:**
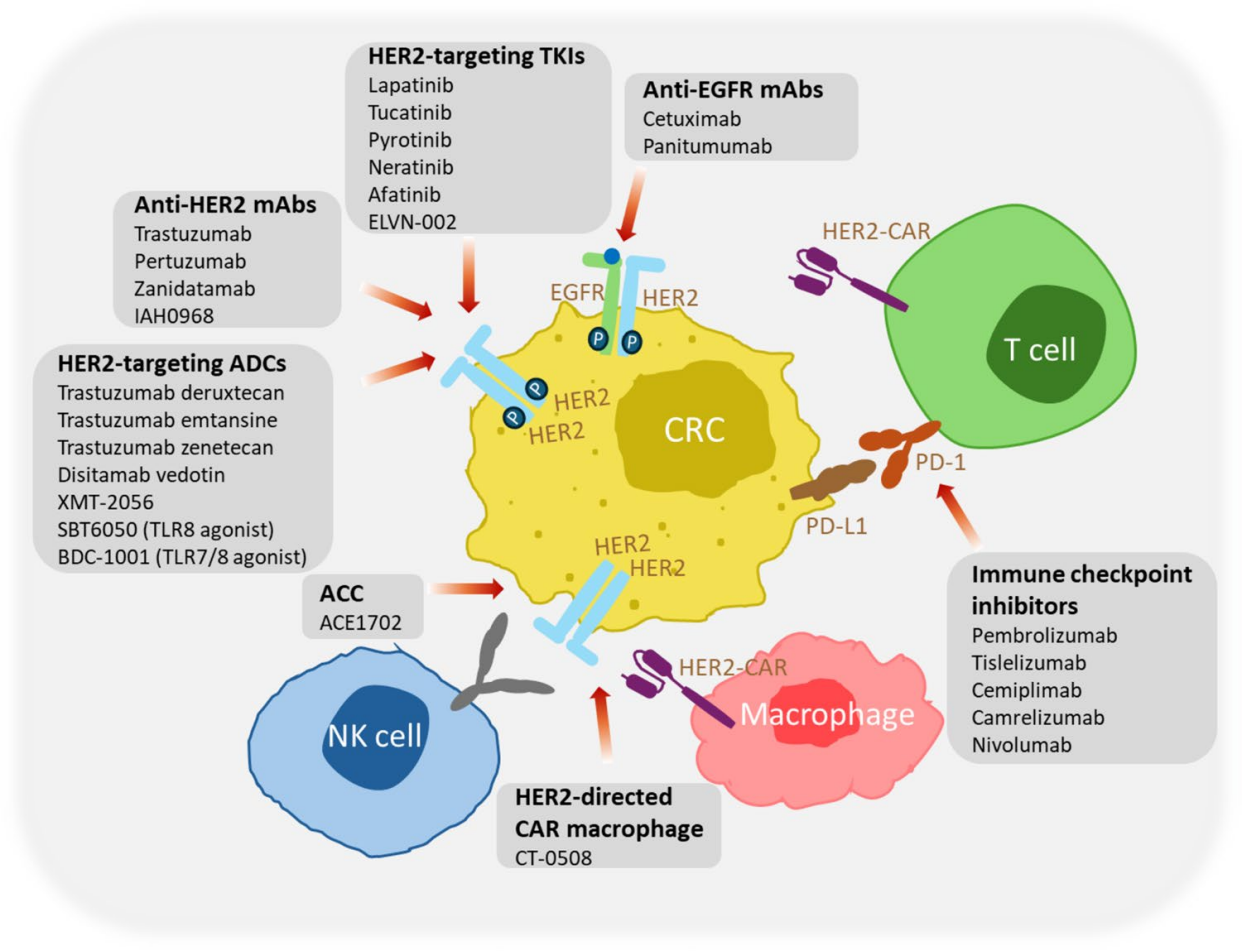
Current HER2-targeting therapies tested for CRC. The figure highlights HER2 and EGFR inhibition using mAbs, TKIs, and ADCs. Immune-based interventions, including immune checkpoint inhibitors, HER2-directed chimeric antigen receptor (CAR) T cells and macrophages, and antibody-cell conjugates (ACC) recruiting natural killer (NK) cells contribute to antitumor responses

Extensive research has firmly established the oncogenic role of HER2 in various human malignancies, including CRC [37–39]. This compelling evidence underscores the potential of therapies targeting HER2 for the treatment of CRC. Antibodies (i.e., trastuzumab, pertuzumab), tyrosine kinase inhibitors (TKIs; i.e., lapatinib, neratinib, tucatinib), antibody drug conjugates (ADCs) like ado-trastuzumab emtansine (T-DM1) and T-DXd, and HER2-targeting immunotherapy are among the current therapeutic agents targeting HER2 in HER2-positive colorectal tumors (Fig. 2) [20, 47–49]. Most of them are still under detailed investigation about the exact mechanisms, safety, and efficacy.

To date, numerous preclinical research and clinical trials including HERACLES-A/B, MYPATHWAY, DESTINY-CRC01, TRIUMPH, and MOUNTAINEER have been conducted to elucidate the efficacy of anti-HER2 regimens. They have provided evidence supporting their clinical benefits in HER2-positive mCRC cases. These trials utilizing therapies such as trastuzumab, lapatinib, pertuzumab, T-DXd, or tucatinib have shown positive outcomes and improved treatment responses in these patients. Anti-HER2 regimens tested for HER2-positive mCRC patients with efficacy outcomes are summarized in Table 1.

**Table 1:** Summary of efficacy outcomes of HER2 therapies in refractory HER2 positive mCRC

Study	Regimen	Phase	N	ORR	Eligibility criteria	Median PFS months (95% CI)	Median OS Months (95% CI)	Trial status^a^	References
Monoclonal antibody (mAb)									
MYPATHWAY^b^ NCT02091141	Trastuzumab plus pertuzumab	II	69	31.9%	HER2 overexpression and/or amplification	4.1 (2.7 to 5.6) months	15.5 (10.3 to 20.9) months	Completed	[40]
TAPUR NCT02693535	Trastuzumab plus pertuzumab	II	28	25%	HER2 amplification or overexpression	17.2 (11.1 to 27.4) weeks	60.0 (32.1 to 102.3) weeks	Recruiting	[41]
TRIUMPH UMIN000027887	Trastuzumab plus pertuzumab	II	17	35%	HER2-positive mCRC, IHC 3 + , *RAS* wt	4.0 (1.4 to 5.6) months	NR	Unknown	[42]
Meric Bernstam et al., 2022 NCT02892123	Zanidatamab	I	26	38%	HER2 IHC 3 + or 2 + /ISH, *KRAS* wt	6.8 (3.5 to 7.8) months	NR	Active, not recruiting	[43]
NCT03185988	Trastuzumab plus irinotecan	II	21	33.3%	HER2 IHC 3 + or 2 + proven by FISH, SISH or CISH, *KRAS/NRAS/BRAF* wt	4.3 (2.7 to 5.9) months	17.9 (11.8 to 24.1) months	Unknown	[44]
Antibody drug conjugates (ADC)									
DESTINY-CRC01 NCT03384940	T-DXd	II	53	45.3%	HER2 IHC 3 + or 2 + /ISH positive, *RAS*/*BRAF* wt, Chemotherapy refractory	6.9 (4.1 to 8.7) months	15.5 (8.8 to 20.8) months	Completed	[45]
DESTINY-CRC02 NCT04744831	T-DXd	II	82	37.8%^c^	HER2 IHC 3 + or 2 + /ISH positive, *BRAF* wt	5.8 (4.6 to 7.0) months^c^	NR	Completed	[11]
HERACLES-B NCT03225937	T-DM1 plus pertuzumab	II	31	9.7%	HERACLES diagnostic criteria by IHC and FISH, *RAS* wt	4.1 (3.6 to 5.9) months	NR	Completed	[46]
NCT04513223	Trastuzumab rezetecan	I	32	46.9%	HER2-expressing advanced/unresectable or metastatic CRC	2.9 (1.5 to 7.1) months^d^	NR	Active, not recruiting	[47]
Tyrosine kinase inhibitor (TKI)-containing regimens									
HERACLES-A NCT03225937	Trastuzumab plus lapatinib	II	32	28%	HERACLES diagnostic criteria by IHC and FISH, *KRAS* wt	4.7 (3.7 to 6.1) months	10.0 (7.9 to 15.8) months	Unknown	[48]
MOUNTAINEER NCT03043313	Tucatinib with or without trastuzumab	II	84	Tucatinib plus trastuzumab: 38.1%, Tucatinib alone: 3.3%	HER2 IHC 3 + or 2 + /ISH positive, *RAS* wt	tucatinib plus trastuzumab: 8.2 (4.2 to 10.3) months, tucatinib alone: NR	Tucatinib plus trastuzumab: 24.1 (20.3 to 36.7) months, tucatinib alone: NR	Completed	[49]
HER2-FUSCC-G NCT04960943	Pyrotinib plus trastuzumab	II	16	50.0%	HERACLES diagnostic criteria for HER2-positive mCRC with at least two prior lines of treatment	7.53 (5.19 to 9.87) months	16.8 (8.72 to 24.87) months	Unknown	[50]
Fu et al., 2023 NCT04380012	Pyrotinib plus trastuzumab	II	18	22.2%	HER2 IHC 3 + or 2 + /SISH or FISH, *BRAF* wt	3.4 (1.8 to 4.3) months	Not reached	Unknown	[51]

*HER2* human epidermal growth factor receptor 2, *ORR* objective response rate, *PFS* progression free survival, *OS* overall survival, *wt* wild-type, *IHC* immunohistochemistry, *ISH *in situ hybridization, *FISH* fluorescent in situ hybridization, *SISH* silver in situ hybridization, *CISH* chromogenic in situ hybridization, *ctDNA* circulating tumor deoxyribonucleic acid, *NR* not reported, *T-DXd* trastuzumab deruxtecan; *T-DM1* trastuzumab emtansine

^a^Status accurate as of 18 November 2024

^b^Patients with HER2 amplification/overexpression and wild-type *KRAS*

^c^Patients who received 5.4 mg/kg T-DXd dose

^d^Time to recurrence (TTR), median range

Currently, there are three different regimens which are recommended in National Comprehensive Cancer Network (NCCN) Clinical Practice Guidelines in Oncology for mCRC patients with *HER2* amplifications [52]. These include T-DXd monotherapy or trastuzumab in combination with either lapatinib or pertuzumab [53–55]. These drugs are originally approved for the treatment of human breast cancers characterized by overexpression of HER2 which occupies around 20–30% of the cases [53, 56]. Now, they are also suggested for CRC patients as well. Individual or combinatorial use with conventional chemotherapeutics or other targeting therapies of these agents have remarkably improved the survival outcome of patients with HER2-positive CRC malignancies [18].

### HER2-targeting antibodies

Trastuzumab (Herceptin^®^) is the first therapeutic humanized monoclonal antibody (mAb) that binds to HER2’s extracellular domain (IV), preventing dimerization and inactivating its intracellular tyrosine kinase domain [57]. It was approved by the Food and Drug Administration (FDA) in 1998 for the treatment of HER2-positive metastatic breast cancer (mBC) [58]. In the MYPATHWAY phase II trial, combined treatment of trastuzumab and pertuzumab showed a good objective response rate (ORR; 31.9%) in *KRAS* wild-type patients [59]. Similar response rates have been also observed in other phase II clinical trials such as TAPUR [41] and TRIUMPH [42] examining the same drug combinations. Although, in TAPUR trial, pertuzumab plus trastuzumab cotreatment does not show an effective antitumor activity in CRC with *HER2/3* mutations, CRC patients with *HER2* amplification exhibited promising response rates to these drugs [41]. Trastuzumab was also tested with a chemotherapy agent, irinotecan, in an open-label phase II study where the patients had *RAS* and *BRAF* wild-type HER2-positive unresectable mCRC (NCT03185988) [44]. Irinotecan, a topoisomerase I inhibitor, was originally made available for purchase in Japan in 1994 for the treatment of a number of malignancies, including ovarian, cervical, and lung tumors [60]. In this clinical trial, the ORR was determined as 33.3% (7/21). Moreover, trastuzumab is being tested in combination with a programmed cell death 1 (PD-1) inhibitor, camrelizumab, in a phase II study (NCT05193292) in patients with HER2-positive CRC. Besides these trials, numerous other active trials are currently investigating the potential of several different combinations or other novel anti-HER2 therapies in treating HER2-positive or *HER2*-mutated CRC (Table 2).

**Table 2:** List of selected ongoing clinical trials of anti-HER2 agents in HER2-altered CRC patients

Study	Study title	Regimen	Anti-HER2 agent	Phase	Patient profile	Trial status^a^
HER2 amplification/overexpression						
TAPUR NCT02693535	Testing the use of FDA-approved drugs that target a specific abnormality in a tumor gene in people with advanced-stage cancer	Trastuzumab plus pertuzumab	mAb	II	HER2 amplification or overexpression	Recruiting
DRUP NCT02925234	The drug rediscovery protocol	Trastuzumab plus pertuzumab	mAb	II	Treatment-refractory HER2-positive mCRC	Recruiting
MOUNTAINEER-03 NCT05253651	A study of tucatinib with trastuzumab and mFOLFOX6 versus standard of care treatment in first-line HER2-positive metastatic colorectal cancer	Tucatinib plus trastuzumab and mFOLFOX6	mAb and TKI	III	HER2-positive mCRC, *RAS* wt	Recruiting
NCT05673512	To evaluate IAH0968 in combination with CAPEOX in HER2-positive metastatic colorectal cancer	IAH0968 plus CAPEOX	mAb	II/III	HER2 IHC 3 + or 2 + FISH, *KRAS, NRAS*, *BRAF* wt	Recruiting
NCT05193292	Camrelizumab combined with trastuzumab and chemotherapy in patients with HER2-positive advanced colorectal cancer	Camrelizumab plus trastuzumab, XELOX, mFOLFOX6, FOLFIRI, mXELIRI and mIRIS	mAb	II	HER2 positivity defined as the colorectal cancer-specific HERACLES diagnostic criteria	Not yet recruiting
NCT05985707	The efficacy and safety of KN026 combination chemotherapy ± KN046 in HER2-positive advanced colorectal cancer and biliary tract cancer as first-line treatment	KN026 plus KN046 and XELOX	Bispecific Ab	II	HER2 IHC 3 + or 2 + /ISH + CRC, *RAS, BRAF* wt	Not yet recruiting
NCT03929666	A safety and efficacy study of ZW25 (zanidatamab) plus combination chemotherapy in HER2-expressing gastrointestinal cancers, including gastroesophageal adenocarcinoma, biliary tract cancer, and colorectal cancer	Zanidatamab plus capecitabine and cisplatin	Bispecific Ab	II	HER2 IHC 3 + or 2 + /ISH + CRC, *RAS, BRAF* wt	Active, not recruiting
UNICORN NCT05845450	Pre-operative targeted treatments in molecularly selected resectable colorectal cancer	Trastuzumab deruxtecan	ADC	II	HER2 IHC 3 + or 2 + /ISH + CRC, pMMR/MSS status	Recruiting
NCT05493683	Disitamab vedotin combined with tislelizumab in advanced HER2 positive colorectal cancer	Disitamab vedotin plus tislelizumab	ADC	II	HER2 IHC 3 + or 2 +	Recruiting
NCT05333809	Pembrolizumab and disitamab vedotin in HER2-expressing metastatic colorectal cancer	Disitamab vedotin plus pembrolizumab	ADC	II	HER2 IHC 3 + or 2 + /ISH + CRC, *RAS, BRAF* wt	Not yet recruiting
NCT05785325	RC48-ADC combined with bevacizumab in HER2-positive advanced colorectal cancer	RC48-ADC plus bevacizumab	ADC	II	HER2 IHC 2 + /FISH positive or IHC3 +	Recruiting
NCT05514717	A study of XMT-2056 in advanced/​recurrent solid tumors that express HER2	XMT-2056	ADC	I	HER2 IHC 3 + or 2 + /ISH + CRC	Recruiting
NCT05382364	Safety and pharmacokinetics of tucatinib (MK-7119) in Chinese participants with cancer	Tucatinib	TKI	I	HER2-positive advanced CRC	Active, not recruiting
NCT05356897	Tucatinib combined with trastuzumab and TAS-102 for the treatment of HER2 positive metastatic colorectal cancer in molecularly selected patients, 3 T Study	Tucatinib plus trastuzumab and TAS-102	TKI and mAb	II	HER2 amplified and *PIK3CA, RAS*, and/or *BRAF*-mutated mCRC	Recently withdrawn, completed
NCT06328738	ELVN-002 with trastuzumab + /​- chemotherapy in HER2-positive solid tumors, colorectal and breast cancer	ELVN-002 plus trastuzumab + /​- chemotherapy	TKI and mAb	I	IHC3 + , IHC2 + /ISH + , NGS amplification by tissue, *RAS, BRAF* wt	Recruiting
NCT04460456	A study of SBT6050 alone and in combination with PD-1 inhibitors in subjects with advanced HER2 expressing solid tumors	SBT6050 plus pembrolizumab and cemiplimab	ADC, immune stimulating	I	Locally advanced or metastatic HER2 IHC 2 + or 3 +	Unknown
NCT04278144	A first-in-human study using BDC-1001 as a single agent and in combination with nivolumab in advanced HER2-expressing solid tumors	BDC-1001	ADC, immune stimulating	I/II	Advanced HER2-expressing solid tumors	Active, not recruiting
VISTA NCT03740256	Binary oncolytic adenovirus in combination with HER2-specific autologous CAR VST, advanced her2 positive solid tumors	HER2 specific CAR T cells plus CAdVEC	HER2 targeting immunotherapy	I	HER2 IHC ≥ 2 +	Recruiting
NCT04660929	CAR macrophages for the treatment of HER2 overexpressing solid tumors	CT-0508	HER2 targeting immunotherapy	I	HER2 overexpression	Active, not recruiting
NCT04319757	ACE1702 in subjects with advanced or metastatic HER2-expressing solid tumors	ACE1702 plus cyclophosphamide and fludarabine	ADC with HER targeting immunotherapy	I	HER2 IHC ≥ 2 +	Recruiting
HER2 amplification/overexpression or mutated *HER2*						
NCT05661357	Disitamab vedotin combined with fruquintinib for mCRC with HER2 expression (HCCSC-C03)	Disitamab vedotin plus fruquintinib	ADC	IV	Advanced CRC with HER2 expression (IHC 1 + , 2 + or 3 +) or mutation detected by NGS	Active, not recruiting
DASH NCT04704661	Testing the combination of two anticancer drugs, DS-8201a and AZD6738, for the treatment of patients with advanced solid tumors expressing the HER2 protein or gene	DS-8201a plus AZD6738	ADC	I	HER2 IHC 1–3 + and FISH	Recruiting
NCT05350917	Study of tislelizumab combined with disitamab vedotin and pyrotinib maleate in HER2-positive or mutated advanced colorectal cancer who failed standard therapy	Tislelizumab plus disitamab vedotin and pyrotinib maleate	ADC	II	HER2 IHC 2 + /FISH positive or IHC3 + or mutation detected by NGS	Not yet recruiting
DETERMINE NCT05786716	Trastuzumab in combination with pertuzumab in adult, teenage/​young adult and pediatric patients with cancers with HER2 amplification or activating mutations	Trastuzumab plus pertuzumab	mAb	II/III	HER2 amplification or activating mutations	Recruiting
TAPUR NCT02693535	TAPUR: Testing the Use of Food and Drug Administration (FDA) approved drugs that target a specific abnormality in a tumor gene in people with advanced-stage cancer	Trastuzumab plus pertuzumab or trastuzumab plus tucatinib	mAb	II	HER2 amplification or overexpression, and specific *HER2* mutations	Recruiting
NCT03457896	Study of neratinib + trastuzumab or neratinib + cetuximab in patients with KRAS/​NRAS/​BRAF/​PIK3CA wild-type metastatic colorectal cancer by HER2 status	Neratinib plus trastuzumab or neratinib plus cetuximab	TKI plus mAb	II	*KRAS/NRAS/BRAF/PIK3CA* wt, mCRC with amplified, non-amplified [wt], or mutated HER2 status	Unknown (was active, not recruiting)
Mutated *HER2*						
DPT01 NCT04639219	A study of T-DXd for the treatment of solid tumors harboring HER2 activating mutations	Trastuzumab deruxtecan	ADC	II	Unresectable and/or metastatic solid tumors with pre-specified HER2 mutations determined by NGS	Active, not recruiting

*mAb* monoclonal antibody, *ADC* antibody–drug conjugate, *TKI* tyrosine kinase inhibitor, *NGS* next-generation sequencing

^a^Status accurate as of 18 November 2024

Targeting HER2 with another humanized monoclonal antibody, pertuzumab, initiates antibody-dependent cellular cytotoxicity (ADCC), cell-cycle arrest, and impaired DNA repair leading to apoptosis. Pertuzumab binds to HER2 extracellular domain II and prevents its dimerization with other HER family receptors, particularly HER2/HER3 heterodimer [61]. Synergistic work between pertuzumab and trastuzumab encouraged several clinical trials which are conducted in patients with advanced HER2-positive CRCs and demonstrated promising clinical benefit for dual HER2 blockade [41, 59, 62, 63]. Trastuzumab is currently being tested in combination with pertuzumab in different clinical trials such as DRUP (NCT02925234) [64] and DETERMINE (NCT05786716) for the cancers with *HER2* amplification. However, this promising profile of drug combinations can be impaired with several other factors. For instance, according to a recent precision medicine molecular case report, a patient from MYPATHWAY trial diagnosed with mCRC harboring *HER2* amplification with concurrent *HER2-del16* splice and *TP53* missense mutations exhibited an aggressive tumor progression and resistance to trastuzumab and pertuzumab combination therapy [65].

Zanidatamab (ZW25) is a bispecific antibody targeting HER2 with biparatopic binding to its ECD4 (juxtamembrane) and ECD2 (dimerization) extracellular domains [43]. An interventional phase I clinical trial of zanidatamab (NCT02892123) conducted in patients with locally advanced (unresectable) and/or metastatic HER2-expressing, *KRAS* wild-type CRC showed a promising clinical response with 38% ORR [43]. PDX models developed from pretreatment or post progression biopsies on the this trial identified amplification of *MET* as a potential mechanism of acquired resistance to zanidatamab which was overcome by MET inhibitors [66]. Moreover, the safety and efficacy of zanidatamab is also being tested in combination with chemotherapy (capecitabine and cisplatin) in HER2-positive CRC (NCT03929666) [67].

IAH0968 is an afucosylated novel mAb targeting HER2 exhibiting similar binding characteristics to trastuzumab but showing enhanced ADCC activity and superior antitumor efficacy [68]. A safety and dose establishing phase I/II trial (NCT04934514) in heavily pretreated patients with *HER2*-amplified metastatic tumors, including colon cancer, showed promising clinical activity and tolerable safety profile. Only one patient out of 18 had a dose-limiting complication (grade 4 arrhythmia) [68]. This led to the phase II/III study of IAH0968 in combination with CAPEOX (oxaliplatin and capecitabine chemotherapy) in HER2-positive mCRC patients who are also *KRAS*, *NRAS,* and *BRAF* wild type (NCT05673512) [69].

KN026 is another novel bispecific anti-HER2 antibody consisting of the heavy chain variable domain of trastuzumab and pertuzumab. It showed a safety profile and promising antitumor activity in patients with HER2-positive advanced gastric or gastroesophageal junction cancer, which led to a phase II clinical trial (NCT05985707) in HER2-positive CRC patients [70].

### Antibody drug conjugates

T-DM1 (Kadcyla^®^) is an ADC of trastuzumab and the microtubule-targeting agent, DM1 combined with a stable linker, MCC [71]. T-DM1 stores all the cytostatic functions of trastuzumab, therefore, targets HER2. The HERACLES-B trial was the first clinical trial which evaluated the efficacy of an ADC for the treatment of HER2-positive chemorefractory mCRC patients [72]. 31 patients were treated with T-DM1 and pertuzumab where they exhibited median PFS of 4.1 months (95%CI 3.6 to 5.9) and ORR of 9.7% (95% CI 0 to 28). Although the study failed to achieve its primary end point for ORR, the anti-HER2 regimen offered remarkable rates of sustained disease control (67.7%, *n* = 21) at the cost of minimal toxicity [46].

In August 2023, the FDA granted breakthrough therapy status to T-DXd (Enhertu^®^) for two groups: patients with unresectable or metastatic HER2-positive (IHC 3+) solid tumors with no other options and those with HER2-positive (IHC 3+) mCRC after at least two prior treatments [73]. T-DXd is a next-generation HER2-targeting ADC combining trastuzumab and a cytotoxic payload (DX-8951f), a topoisomerase I inhibitor with a maleimide peptide linker [74]. The antitumor activity and safety of T-DXd was investigated in DESTINY-CRC01 open-label phase II study in patients with mCRC progression after ≥2 prior regimens [45]. 53 HER2-positive patients received 6.4 mg/kg T-DXd for every 3 weeks and exhibited ORR of 45.3% (95% CI 31.6–59.6) with 15.5 months overall survival (95% CI 8.8–20.8). After two or more prior treatments, such as irinotecan and other HER2-targeting drugs, patients with HER2-positive mCRC responded strongly and durably to T-DXd therapy [45, 75]. In another phase II T-DXd trial, DESTINY-CRC02, HER2-positive, *RAS* wild-type, or mutant mCRC patients received two different doses of T-DXd as 5.4 (*n* = 82) and 6.4 mg/kg (*n* = 40). This study revealed that the numerical responses of patients receiving the lower 5.4-mg/kg dosage of T-DXd were higher than those receiving the higher 6.4-mg/kg dose. The confirmed ORR for the 5.4 mg/kg dose-receiving group was 37.8% (95% CI 27.3–49.2%), whereas it was 27.5% (95% CI 14.6–43.9%) in the other group receiving higher dose. Anti-tumor efficacy was seen regardless of *RAS* mutation status of the patients in the 5.4 mg/kg arm as 39.7% with *RAS* mutations and 28.6% without *RAS* mutations. T-DXd is also currently being tested in a phase II window-of-opportunity umbrella platform trial (UNICORN; NCT05845450) in patients with HER2-positive non-metastatic resectable CRC. Moreover, a phase I/Ib study (DASH; NCT04704661) is currently testing the safety and tolerability of a combinatorial treatment of T-DXd and AZD6738 (ceralasertib), an Ataxia telangiectasia and Rad3 related (ATR) kinase inhibitor, in CRC patients with a change in the *HER2* gene or protein. Additionally, the ongoing DPT01 basket study (NCT04639219) is evaluating the T-DXd efficacy and safety in CRC patients with HER2-activating mutations.

A meta-analysis to assess the efficacy of HER2-targeted treatment regimens in HER2-positive mCRC patients revealed that T-DXd had the most effective disease control rate (DCR) (>80%), followed by T-DM1 plus pertuzumab (77.42%) and trastuzumab plus tucatinib (71.43%) [76]. Additionally, T-DXd has been intensively tested in HER2-low solid tumors including breast [77], gastric [78], and colorectal cancers [45, 78]. Although T-DXd exhibited a promising clinical activity in HER2-low breast and gastric tumors, the response in HER2-low mCRC tumors were quite insignificant.

Trastuzumab rezetecan (SHR-A1811) is a novel HER2-directed ADC composed of trastuzumab, a stable and cleavable linker, and a novel topoisomerase I inhibitor payload (SHR9265) [79]. An ongoing phase I clinical trial (NCT04513223) is assessing the tolerability, safety, pharmacokinetics, and immunogenicity of SHR-A1811 in HER2-expressing advanced/unresectable or mCRC. The preliminary results demonstrated that 32 HER2-positive mCRC patients had ORR of 46.9% (95% CI 29.1–65.3) and TTR median of 2.9 months (95% CI 1.5–7.1), indicating an acceptable safety profile and promising clinical activity in these patients who also require further investigation [47].

Disitamab vedotin (RC48) is another newly developed HER2-targeting ADC composed of hertuzumab coupling monomethyl auristatin E (MMAE) via a cleavable linker [80]. Patients with HER2-positive CRC had demonstrated promising therapeutic responses to disitamab vedotin in early phase trials. Combination therapies of disitamab vedotin with immune checkpoint inhibitors, PD-1, such as pembrolizumab (NCT05333809) or tislelizumab (NCT05493683 and NCT05350917), or with an oral inhibitor of vascular endothelial growth factor (VEGF) receptor, fruquintinib (NCT05661357), are being studied in terms of safety and efficacy in HER2-expressing (amplified/overexpressed or mutated) mCRC patients [62, 81, 82].

XMT-2056 is another ADC targeting a novel HER2 epitope and locally activating the stimulator of interferon gene (STING) pathway. Its safety and preliminary efficacy is being tested in a phase I trial (NCT05514717) in HER2-positive CRC patients [83].

### Tyrosine kinase inhibitor-containing regimens

Tucatinib (Tukysa^®^) is an oral tyrosine kinase inhibitor for kinase domain of HER2 with > 50-fold selectivity over EGFR, which also crosses the blood–brain barrier [84]. Safety and pharmacokinetics of tucatinib is being tested in HER2-positive advanced CRC patients from China in a phase I trial (NCT053882364). Moreover, combination therapy with tucatinib and trastuzumab significantly enhanced the anticancer efficacy in xenograft models of colorectal, gastric, breast, and esophageal malignancies [84, 85], which led to several clinical trials. The MOUNTAINEER trial evaluated the safety and efficacy of trastuzumab plus tucatinib in patients with chemotherapy-resistant, HER2-positive, *RAS* wild-type, unresectable, or mCRC, which were randomized to receive tucatinib alone or combination treatment. The final results indicated that 86 patients treated with dual therapy and 30 in the tucatinib cohort exhibited ORRs of 38.1% and 3.3%, respectively [49]. With that, on January, 2023, FDA approved this combinatorial therapy as the first anti-HER2 regimen for HER2-positive mCRC patients. These results led to different ongoing studies such as a follow-up study called MOUNTAINEER-03 (NCT05253651), testing tucatinib and trastuzumab combination with standard treatment regimens using chemotherapy like mFOLFOX6 and other targeted therapies for HER2-positive, *RAS* wild-type mCRC patients [86]. Furthermore, another phase II study (NCT05356897) testing whether tucatinib combined with trastuzumab and TAS-102, oral chemotherapy approved for the treatment of mCRC, exhibited results of shrinking tumors of HER2-positive mCRC patients with one of the following gene mutations: *PIK3CA, KRAS, NRAS, or BRAF* V600*.*

Lapatinib is an oral dual tyrosine kinase inhibitor of EGFR and HER2 binding to the ATP-binding site of the intracellular domain of the receptor [87]. HERACLES-A clinical trial was the first proof-of-concept, phase II study assessing the efficacy and safety of lapatinib and trastuzumab combination therapy in patients with *KRAS* wild-type, chemorefractory HER2-positive mCRC. 35 patients with long-term follow-up (6.7 years) showed a median PFS of 4.7 months and an ORR of 28% [48].

Pyrotinib is an irreversible dual pan-ErbB receptor TKI developed for the treatment of HER2-positive advanced solid tumors receiving its first global approval for the treatment of HER2-positive breast cancer [88]. A multicenter phase II trial (NCT04380012) tested the efficacy and safety profile of pyrotinib and trastuzumab combination for the treatment of HER2‐positive recurrent/metastatic CRC. Out of 18 *BRAF* wild-type patients, 4 had a partial response with an ORR of 22.2%. For both *BRAF* and *RAS* wild-type patients, the ORR was 33.3%, indicating that combination treatment exhibited promising antitumor effects and a tolerable safety profile [51]. Another phase II trial called HER2-FUSCC-G tested the efficacy and long-term safety of pyrotinib and trastuzumab combination for patients with mCRC who had undergone at least two prior lines of treatment. ORR was measured as 50% in the overall population of 16 patients, while it was 57.1% in *RAS* wild-type patients (*n* = 14). 5 (31.3%) patients reported grade 3 treatment-emergent adverse effects (TEAEs), and there was no death reported as of yet [50].

Neratinib (NerlynxTM^®^) is irreversibly inhibiting phosphorylation of EGFR family receptors except HER3 and downstream pathways including ERK/MAPK and Akt [89]. Neratinib efficacy and safety was assessed in a phase II (NCT03457896) study together with trastuzumab or cetuximab in patients with quadruple wild-type (*KRAS/NRAS/BRAF/PIK3CA)* mCRC based on *HER2* status [90]. Preliminary data exhibit an ORR of 33% in all patients who received at least one dose of anti-EGFR therapy. Moreover, neratinib and cetuximab combination was moderately tolerated with some expected side effects such as diarrhea and rash. The final situation of the study is unknown to date as it does not release any specific data about the patients receiving trastuzumab and neratinib combination.

Finally, ELVN-002 as a novel irreversible inhibitor of HER2 with a > 100-fold selectivity over EGFR has been recently developed [91]. Its safety and tolerability profiles are being tested in an ongoing study (NCT06328738) in combination with trastuzumab and chemotherapy in patients with advanced-stage HER2-positive CRC.

### HER2-targeting immunotherapy

Complex nature of CRC with HER2 amplification/overexpression or mutation has enabled therapeutic expansion including the development and use of targeted therapies and immunotherapy. Therefore, different strategies inducing immune system together with HER2-targeting are also emerging as novel treatment options for HER2-positive CRC.

Pertuzumab zuvotolimod (SBT6050) and BDC-1001 are two novel immune-stimulating ADCs, which are currently being tested in clinical trials. SBT6050 combines pertuzumab as a HER2-directed monoclonal antibody with a selective small molecule toll-like receptor 8 (TLR8) agonist which aims to activate myeloid cells, including macrophages and dendritic cells (DCs), and NK and T cells in HER2-positive tumors including CRC. The safety and tolerability of SBT6050 is currently being evaluated as a single agent and in combination with checkpoint inhibitors such as cemiplimab and pembrolizumab targeting PD-1 (NCT04460456) [92]. Similarly, BDC-1001 acts through incorporation of trastuzumab and a TLR7/8 agonist with a non-cleavable linker in HER2-expressing solid tumors including CRC. A first-in-human study (NCT04278144) assesses the safety and tolerability together with a preliminary efficacy profile of BDC-1001 as a single agent and in combination with nivolumab, another PD-1 inhibitor, in HER2 expressing advanced malignancies [93].

Another promising approach is adoptive cell-transfer-based immunotherapy using chimeric antigen receptor (CAR) T cells, which are a novel type of cellular immunotherapies [94]. Although CAR T therapies exhibited promising success in targeting hematological malignancies, they struggle in the context of solid tumors [95]. In this case, oncolytic viruses (OVs) can help CAR T cells overcome some of the immunosuppressive mechanisms caused by tumor microenvironment. According to a recent study, a binary oncolytic/helper-dependent adenovirus (CAdVEC) lyses tumor cells and locally expresses the proinflammatory cytokine IL-12 in humanized mouse models [96]. This led to a first-in-human phase I clinical trial (NCT03740256). The study aims to expand HER2 CAR T cells at primary tumor sites and metastasized tumors with the help of intratumoral injection of CAdVEC inducing a proinflammatory tumor microenvironment [96]. Another phase I study (NCT04660929) is currently investigating the safety and tolerability of HER2-directed CAR macrophages (CarM) therapy, CT-0508, combined with pembrolizumab in HER2-positive tumors including CRC [97].

In addition to CAR technology, Antibody-Cell Conjugation (ACC) technology has been also shown to provide an effective platform for arming immune cells with cancer-targeting antibodies [98]. ACE1702 is a novel off-the-shelf trastuzumab-armed NK cell therapy containing a novel endogenous CD16-expressing oNK cell line (oNK) [99]. An ongoing phase I study (NCT04319757) is evaluating the safety, tolerability, pharmacokinetics, pharmacodynamics, and preliminary efficacy of ACE1702 in patients with advanced or metastatic HER2-expressing tumors, including CRC.

## Conclusion remarks and future perspectives

*HER2* is gaining recognition as a significant biomarker in a specific subset of CRC patients, especially at advanced stages. It can also function as an adverse indicator of effectiveness of EGFR-targeted therapies in individuals with mCRC and be used as a potential target for treatment in HER2-positive mCRC patients who are wild type for *KRAS* and *BRAF*. Although there are limited therapeutic options available for mCRC patients having HER2 amplification or overexpression, there are a growing number of FDA-approved drugs and many ongoing clinical trials using TKIs, ADCs, antibodies, and immune therapy targeting HER2. However, further research is crucial to delve into the intricate mechanisms underlying resistance development to anti-EGFR therapies in HER2-positive metastatic colorectal cancers. This exploration should encompass a comprehensive investigation into the genetic alterations, signaling pathways, and tumor microenvironment interactions that drive resistance, aiming to elucidate the specific molecular determinants and their interplay. Such detailed understanding is pivotal for devising more effective and personalized therapeutic strategies tailored to overcome resistance challenges and enhance treatment outcomes in this patient population.
